# Training protocol for probabilistic Pavlovian conditioning in mice using an open-source head-fixed setup

**DOI:** 10.1016/j.xpro.2021.100795

**Published:** 2021-09-03

**Authors:** Panna Hegedüs, Anna Velencei, Claire-Hélène de Belval, Julia Heckenast, Balázs Hangya

**Affiliations:** 1 Lendület Laboratory of Systems Neuroscience, Institute of Experimental Medicine, Budapest, Hungary; 2 János Szentágothai Doctoral School of Neurosciences, Semmelweis University, Budapest, Hungary; 3 Interdisciplinary Masters’ in Life Sciences, Ecole Normale Supérieure, Paris, France

**Keywords:** Behavior, Model Organisms, Neuroscience

## Abstract

High throughput, temporally controlled, reproducible quantitative behavioral assays are important for understanding the neural mechanisms underlying behavior. Here, we provide a step-by-step training protocol for a probabilistic Pavlovian conditioning task, where two auditory cues predict probabilistic outcomes with different contingencies. This protocol allows us to study the differential behavioral and neuronal correlates of expected and surprising outcomes. It has been tested in combination with chronic *in vivo* electrophysiological recordings and optogenetic manipulations in ChAT-Cre and PV-Cre mouse lines.

For complete details on the use and execution of this protocol, please refer to Hegedüs et al. (2021).

## Highlights

• We provide a training protocol for a probabilistic Pavlovian conditioning task in mice
• Two auditory cues predict probabilistic outcomes with different contingencies
• Possible to combine with chronic *in vivo* electrophysiology and optogenetics
• Ideal for testing behavioral and neural correlates of expected and surprising outcomes

## Graphical abstract

**Figure:**
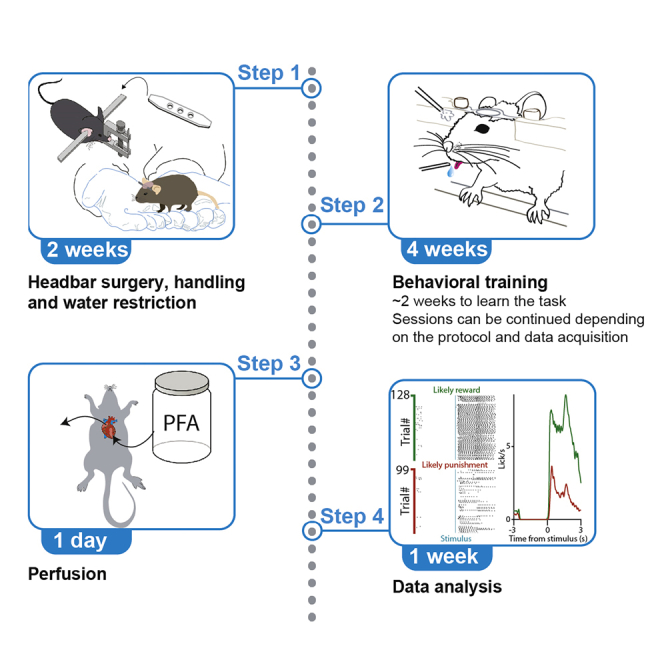

## Before you begin

Pavlovian or classical conditioning is an associative learning paradigm. As a hallmark, the outcome does not depend on the actions of the performing agent, as in the case of Ivan Pavlov’s famous experiment, in which his dog formed an association between a previously neutral auditory stimulus and food reward (Fanselow and Poulos, 2005). This allows the experimenter to have full control over the outcome contingencies, making this task ideal for probing the neural mechanisms underlying the processing of probabilistic outcomes (Pearce and Hall, 1980; Schultz et al., 1997). This Pavlovian training protocol was tested in combination with extracellular tetrode recordings (Hegedüs et al., 2021) and optogenetic tagging of neurons. We confirmed that it is suitable for optogenetic manipulations and fiber photometry experiments as well. Although not tested, we believe that it would also allow investigation of the effects of pharmacological or chemogenetic manipulations on associative learning. We further envision that, with an addition of a head plate designed for imaging window surgeries, it could be used in combination with two photon imaging. Before presenting the training protocol, we describe the preparatory phase of the experiment including animal welfare, headbar surgery and proper handling of mice.

### The training setup

**Figure 1:**
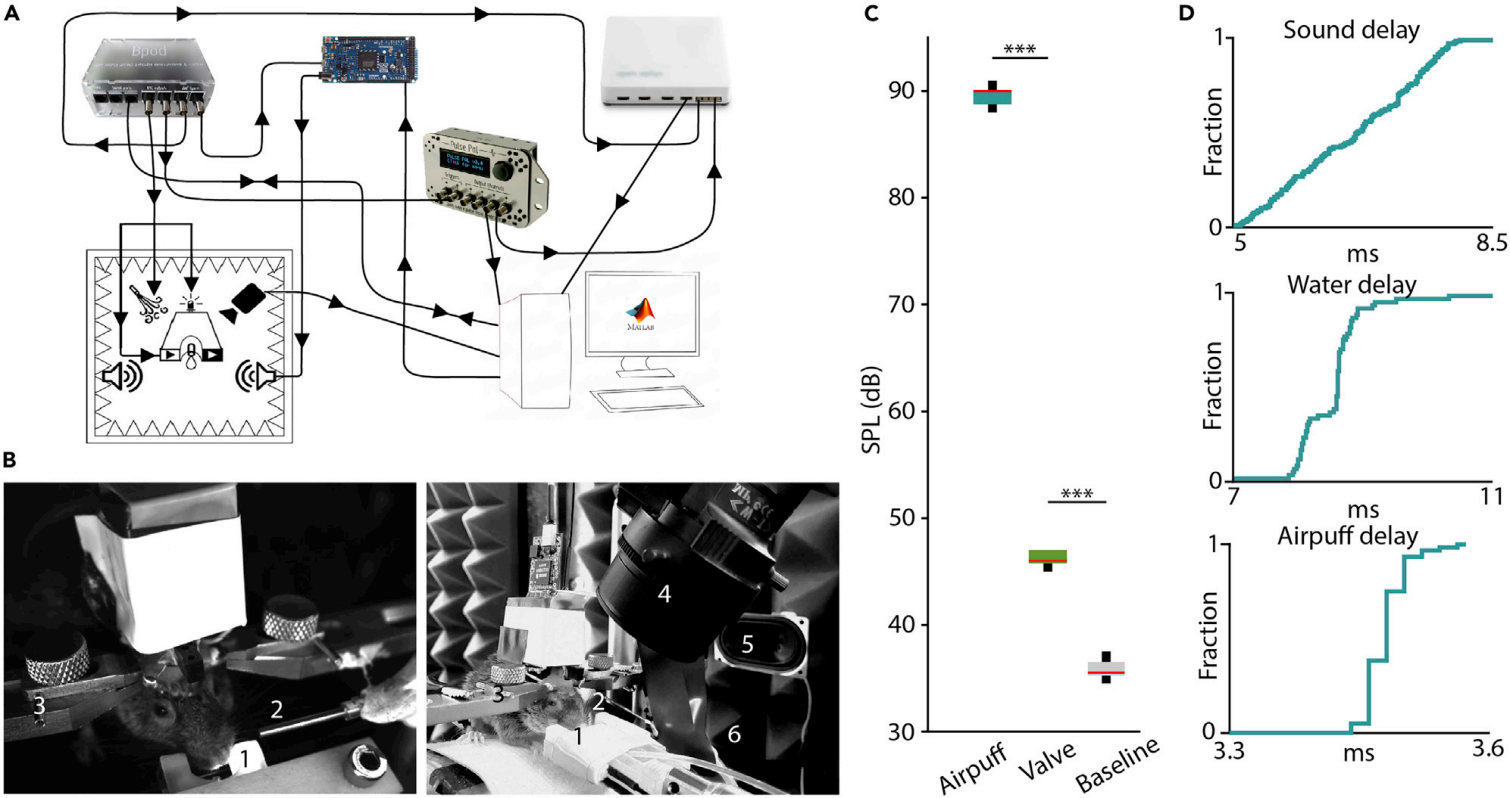
The experimental setup (A) Schematic diagram of the setup. Modified from (Solari et al., 2018). (B) Photos of mice performing the auditory Pavlovian conditioning task. 1, lick port; 2, tubing for air puff; 3, headbar holders; 4, camera; 5, speakers; 6, sound absorbing foams. (C) Sound pressure levels associated with the flow of air at air-puff punishment, the click sound of the solenoid valve at reward presentation and background noise. Box-whisker plots show median, interquartile range and non-outlier range. ∗∗∗, p < 0.001, Mann-Whitney U-test. (D) Delay measurements for sound cues, water and air puff delivery. Underlying data are from refs. (Solari et al., 2018; Hegedüs et al., 2021).

The training apparatus consists of a sound-attenuated training chamber, a head-fixation platform, an open source behavior control unit (Bpod, Sanworks), peripheries for interacting with the animal (lick port, water and air delivery systems, speakers to provide sound stimuli), a camera, optional open source stimulator unit (PulsePal, Sanworks (Sanders and Kepecs, 2014)) and data acquisition board (Open Ephys (Siegle et al., 2017)), and a PC (Figures 1A and 1B). The custom-built sound-insulated Faraday-cage minimizes environmental noise (Figure 1C) and electric noise during the experiment. In the center of the box, an adjustable platform is placed along with two headbar holders to keep the mice’s head in a fixed position during the training sessions. A lick port delivering water reward and a cannula delivering air puff as punishment are adjusted to the animal’s position. A camera is placed at the top right corner of the box and speakers for delivering auditory cues are mounted on the sidewalls of the box. The behavioral protocol is controlled via an open source closed-loop finite state machine (Bpod, Sanworks, see Figures 1A and 1B). This setup allows us to train mice on high-precision behavioral protocols with temporally controlled stimuli (Figure 1D). Details of this open source multipurpose training platform were published in (Solari et al., 2018).

**CRITICAL:** Regularly calibrate valve opening durations to deliver the right amount of water rewards. It is possible to calibrate sound pressure levels of auditory stimuli as well. See (Solari et al., 2018) for details.

### Animal welfare

Our experiments were carried out using ChAT-Cre, PV-Cre and 3×Tg-AD Bl6 (Oddo et al., 2003) young adult (3–6 months) and aged (12–21 months) mice of both sexes.

**CRITICAL:** Before the experiment, ensure that animal care and all experiments are conducted according to the relevant ethical guidelines, reviewed and approved by your institute’s animal welfare and ethical committee, as well as all necessary regulatory agencies.

### Headbar surgery

**Timing: 1 h + 1 week recovery period**

We provide a description of our surgical protocol for mounting a headbar, which is used for head fixation while training mice in the head-fixed setup. We use titanium headbars weighing 0.27 g (dimensions, 19 mm × 3 mm × 1 mm); however, we expect other headfixation systems to provide similar results.

1. Put the mouse on the scale and measure its pre-surgery weight.

2. General anesthesia
  a. Briefly place the mouse in an anesthesia induction chamber (custom-built or commercial) to induce anesthesia by isoflurane inhalation.
    **CRITICAL:** Continuously monitor breathing during the induction.
  b. Inject a mixture of ketamine-xylazine intraperitoneally (83 mg/kg and 17 mg/kg respectively, dissolved in saline).
  c. Confirm the loss of nociceptive reflexes by pinching the paws/tail with surgical tweezers.
  d. Monitor breathing and nociceptive reflexes throughout the surgery.

3. Shave the scalp. It is recommended to apply a small amount of water on the fur before shaving to limit the spread of potential airborne allergens.
4. Disinfect the scalp skin with Betadine.
5. Inject Lidocaine (or other approved local anesthetic) subcutaneously to achieve local anesthesia of the scalp skin and connective tissue.
6. Fix the animal’s head in the stereotaxic frame.
7. Cover the eyes with eye ointment (e.g., Corneregel) to protect them from dehydration and strong light.
8. Remove the skin, connective tissue and periosteum from the calvaria using surgical scissors, forceps and scraper. Optionally, make small scratches on the skull with a scalpel to increase the surface for better adhesion of the dental cement.

**CRITICAL:** Make sure that the skull is perfectly clean and dry. Tissue debris can lead to improper adhesion of the surgical cement and detachment of the implant.

9. Level the skull along both the lateral and antero-posterior axes. We recommend using two symmetrical points over the parietal plates, 2 mm from the sagittal suture for lateral leveling (Király et al., 2020).
10. Cover the calvaria surface with high-adhesive dental cement (e.g., Metabond).

**CRITICAL:** Most high-adhesive cements should be kept and mixed on ice to aid polymerization.

11. Mount the headbar on the top of the skull by using acrylic powder and liquid resin (e.g., from Lang Dental). We recommend positioning the headbar over or right in front of Bregma. Implanting a small wood stick holder can help positioning the head of the mouse during head-fixation.
12. Once the dental cement is cured, remove the mouse from the stereotax.
13. Place the mouse on the scale and measure its weight, now with the implant mounted.
14. Inject analgesic according to your approved protocol (e.g., buprenorphine, BupaQ).
15. We recommend injecting 0.5 mL saline subcutaneously to prevent dehydration. It is recommended to distribute large amounts to multiple injection sites.
16. Put the mouse in its homecage. Place the cage on a heating pad until the mouse wakes up to prevent hypothermia.

***Note:*** Animals were single-housed from the day of the surgery during the entire course of the experiment to prevent aggression and damage to the implants, and to allow proper control over water intake.

***Note:*** Avoid low cage tops that can block the movement of implanted mice. Cage tops can also be used upside down if they do not leave enough space for the implanted mice.

### Handling, water restriction

**Timing: 15–20 min / day for 1 week**

**Figure 2:**
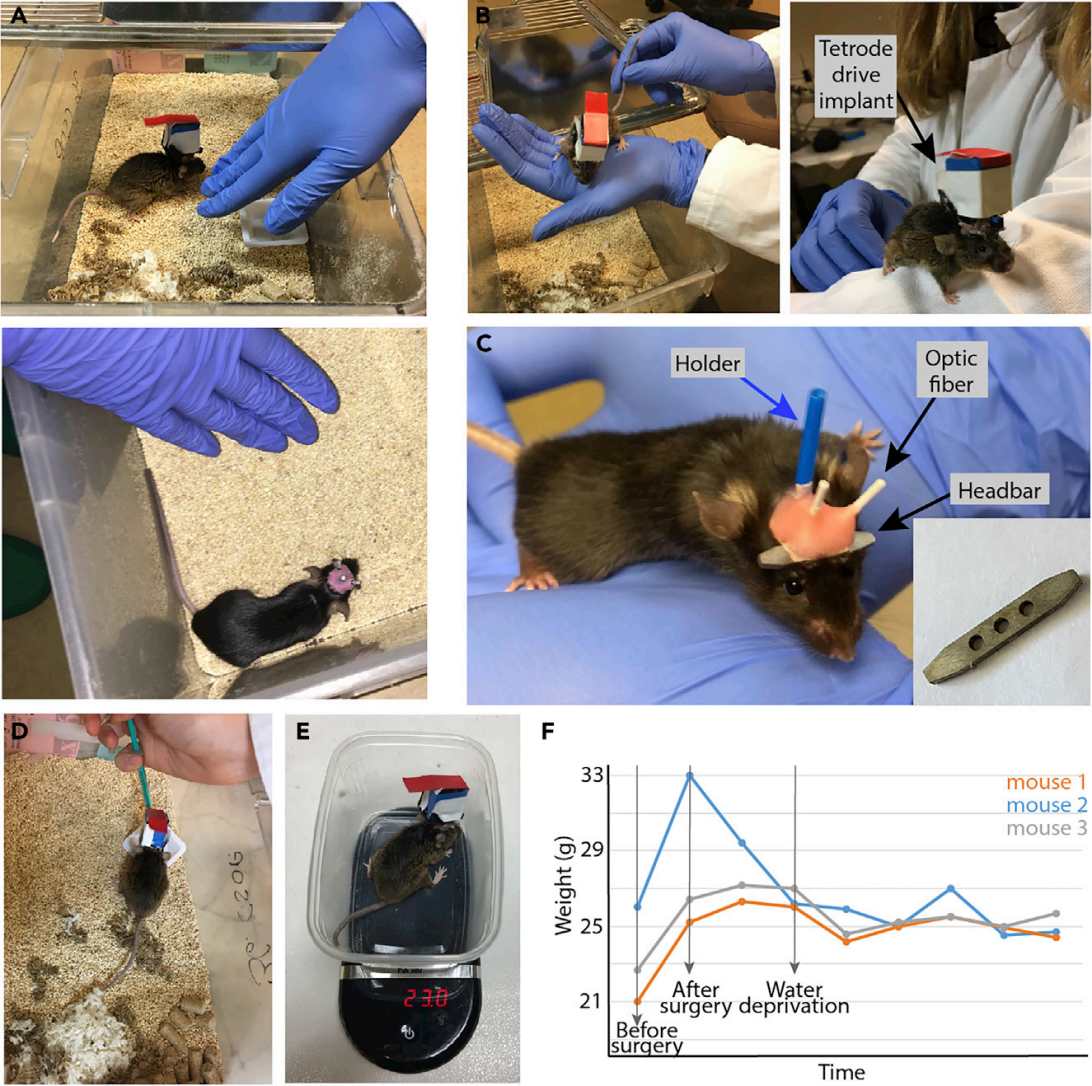
Animal handling (A–C) Different phases of handling mice: the experimenter putting one hand in the cage (A), lifting the mouse (B) and letting it explore (C). Note that the mouse in panel B was implanted with a moveable tetrode drive besides the headbar, while the mouse in panel C had fixed bilateral optic fiber implants. Note also that lifting the mouse by the tail should be avoided; however, gently holding the tail during lifting can prevent the mouse from falling. (D) Provide water in a weigh boat or equivalent container. (E) Measure the weight daily. (F) Mice drop weight at the beginning of water restriction, but the weight loss should be moderate, and the weight should stabilize after a few days and remain stable throughout the experimental phase.

After surgery, mice are allowed a 1-week recovery period. Handling and water restriction of mice starts 1–2 weeks prior to the training protocol. Animals are handled and habituated to human touch on a daily basis. Below, we provide a short description of our handling protocol (see also Figures 2A–2C).

**Pause point:** It is possible to include longer delays between headbar surgery and start of handling and water restriction. However, please note that if the training is combined with recording, then extending this delay could result in decreased signal quality.

17. Transport the animals to the behavioral testing room or facility a few days prior to the beginning of handling (typically ∼1 week after surgery).
18. The experimenter opens the cage in a way that half of it is still covered by the top and places his/her gloved hand into the cage and allows the mice to sniff it or climb onto it.
19. The experimenter lifts the mouse and places it onto his/her open palm a little above the cage; this way the animal starts to get used to human touch but still has the option to climb off.
20. If the animal is not trying to escape from the experimenter, he/she should lift the mouse and let it explore and sniff the experimenter’s hand.
21. Measure the weight of the mouse.
22. Reward the animal with a snack (e.g., sunflower seed) after the handling. We found that mice became used to human touch sooner when the handling process was associated with food reward, in spite of the fact that no food restriction was applied.
23. Water restriction protocol can be started in parallel with handling (Figures 2D–2F). Place a small weigh boat into the cage and fill it with 1 mL of water daily (1–1.5 mL for aged mice), measured with a syringe. Below we provide a list of behavioral and appearance changes that can be signs of dehydration; observing these signs at any time, the daily amount of water should be raised, or the water restriction should be suspended. Signs of severe dehydration in mice:
  a. After pinching the skin over the back, the skin remains bunched up
  b. Mice are weak and having trouble with gripping the cage bar
  c. Mice are curled up in the corner and barely moving or exploring
  d. Shrunken or recessed eyes
  e. Fuzzy facial and back fur

**CRITICAL:** The weight of the animals should be measured daily, and their behavior and potential signs of dehydration should be monitored regularly.

## Key resources table

**Table:** 

REAGENT or RESOURCE	SOURCE	IDENTIFIER
**Experimental models: organisms/strains**		

ChAT-IRES-Cre mice	https://www.jax.org/	Cat#006410
PV-Cre mice	https://www.jax.org/	Cat#008069
3×Tg-AD mice	https://www.mmrrc.org/	Cat#34830-JAX

**Software and algorithms**		

MATLAB R2016a	https://mathworks.com/	R2016a
Algorithms for behavioral analysis	https://github.com/hangyabalazs/VP_data_analysis	Commit#6308804
Algorithms for closed loop behavioral control	https://github.com/hangyabalazs/Bpod_r0_5	CuedOutComeTask
Code for simple data analysis of lick responses and neural recordings	https://github.com/hangyabalazs/CellBase	ultimate_psth.m

**Chemicals, peptides, and recombinant proteins**		

Isoflurane	https://vetcentre.com/	N/A
Ketamine	https://vetcentre.com/	N/A
Xylazine	https://vetcentre.com/	N/A
Lidocaine	Local pharmacy	N/A
Betadine	Local pharmacy	N/A
Buprenorphine	http://www.richter-pharma.com/	N/A
Promethazine	https://vetcentre.com/	N/A
Paraformaldehyde	https://taab.co.uk/	Cat#P001

**Other**		

Standard surgical scissor	https://www.finescience.com/en-US/	Cat#14060-10
Surgical forceps	https://www.finescience.com/en-US/	Cat#11151-10
Scraper	https://www.finescience.com/en-US/	Cat#10075-16
Jet Set-4 Denture Repair Powder and Liquid	https://www.langdental.com/	N/A
Metabond dental cement	http://www.parkell.com/	Cat#S380
Headbar	Custom made	N/A
Headbar holders	Custom made	N/A
Sound-attenuated training chamber	https://github.com/hangyabalazs/Rodent_behavior_setup/	Commit#85897d1
Lick port	https://www.shapeways.com/	N/A
Infrared sensor and emitter	https://www.digikey.com/	Cat#480-1958-ND and Cat#480-1969-ND
Speakers	https://www.digikey.com/	Cat#668-1447-ND
Camera	https://www.flir.eu/iis/machine-vision/	Cat#FL3-U3-32S2M-CS
Bpod	https://sanworks.io/	Cat#1027
Teensy 3.2	https://www.pjrc.com/teensy/	N/A
PulsePal (if photostimulation is performed)	https://sanworks.io/	Cat#1102
Optogenetics Kit (if photostimulation is performed)	https://www.thorlabs.com/newgrouppage9.cfm?objectgroup_id=6148; light source alternative: laserglow.com	N/A
Data Acquisition System (if recording is performed)	https://open-ephys.org/acquisition-system/starter-kit	Version#2.4
Plastic tubing	https://www.thermofisher.com/	Cat#8001-0102 and Cat#8001-0204
Polyethylene tubing	https://www.warneronline.com/	Cat#64-0755/PE-160
Feeding needle	https://www.finescience.com/en-US/	Cat#18060-20
Bulldog serrefine	https://www.finescience.com/en-US/	Cat#18050-28
Bone rongeour	https://www.finescience.com/en-US/	Cat#16012-12

## Step-by-step method details

### Behavioral training

**Timing: 20–60 min / day for 6–8 days for the full training protocol**

**Timing: 20 min for step 2**

**Timing: 30 min for step 3**

**Timing: 40 min for step 4**

**Timing: 40 min for step 5**

**Timing: 50 min for step 6**

**Timing: 60 min for step 7**

**Figure 3:**
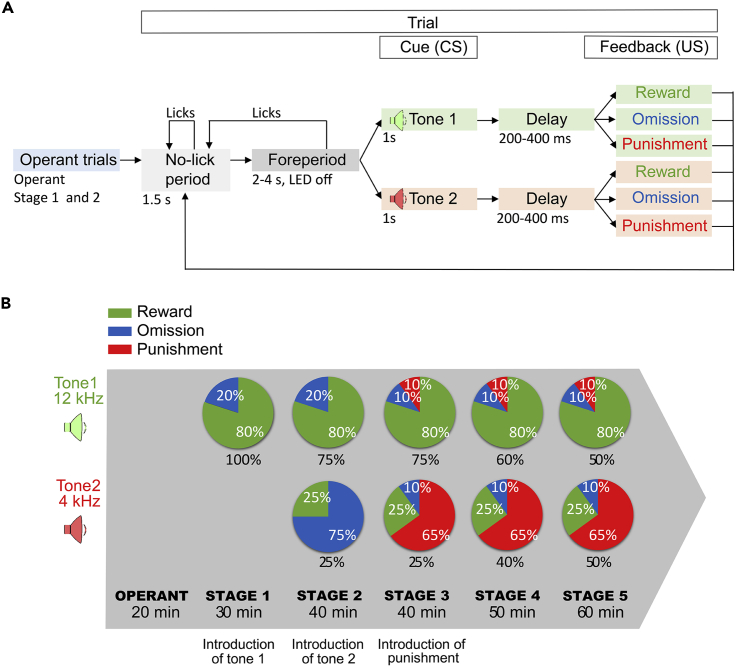
The probabilistic Pavlovian conditioning task (A) Flowchart indicating the states of the task. (B) Schematic diagram of reinforcement contingencies during different training phases.

We provide a detailed description of the training protocol from the beginning to the point when the mice have learned the task (Figures 3A and 3B). We use a 1 training session/day protocol, i.e., each training step we describe here should be performed on consecutive days. The probabilistic training protocol consists of two cues and associated reinforcement; one cue predicts likely reward (80% reward, 10% punishment, 10% omission) and the other cue predicts likely punishment (25% reward, 65% punishment, 10% omission).

***Note:*** These contingencies were calibrated to keep mice motivated on the task. We found that mice often stop licking the water spout when applying a higher ratio of air-puff punishments.

***Note:*** It takes approximately 1 week for young adult animals to reach Stage 5 on the task and then an additional week to achieve stable, good performance. Nevertheless, behavioral sessions of the full task can be continued beyond this period for recording or other purposes based on the experimental design. We observed stable performance for 2–3 months, beyond which we have no experience.

1. Initial steps (perform these before each training session)
  a. Turn on the computer, plug in the speakers, launch Matlab and Bpod. If combined with recording, start the data acquisition system (e.g., Open Ephys; see refs. (Hangya et al., 2015; Laszlovszky et al., 2020)). If combined with optogenetic tagging (Lima et al., 2009; Kvitsiani et al., 2013; Hangya et al., 2015; Széll et al., 2020), turn on the laser.
  b. Make sure that the water tank used for reward is filled.
  c. Clean the head-fixation platform with 70% ethanol.
  d. Remove the air bubbles from the water tube by opening the water valves through Bpod. Make sure to collect the water and thus keep the setup dry.
  e. Put the mouse onto the head-fixation platform of the setup. If combined with recording and/or optogenetics, connect the cables and/or patch chords as appropriate for your system.
  f. Carefully restrain the head of the animal.
  g. Adjust the position of the licking spout with an xyz-stage so that it is close to but not touching the animal’s snout.

**CRITICAL:** Make sure that the licking spout is well-positioned, within convenient range of the animal’s tongue. Too close, too far, and asymmetric positions critically impact behavioral performance. Using a dentist’s mirror tool can aid optimal positioning.

**CRITICAL:** Make sure that air has been completely removed from the water delivery system.

2. Habituation to head restraint (see problem 1), learning to lick for water (Operant phase)
  a. In a brief operant phase, the mouse receives water reward (3–5 μL) every time it licks the water spout (see problem 2).

**CRITICAL:** If the mouse appears distressed, is vocalizing or trying to escape, terminate the protocol and attempt to repeat another time.

3. Introducing the cue that predicts likely reward (Stage 1)
  a. The animal performs 20 operant trials (as in the Operant phase), then the reward predicting cues start.
  b. Each trial starts with a no-lick period, in which the mouse has to withhold licking for 1.5 s. This is followed by a variable foreperiod, randomized between 2 and 4 s.
  c. Then, a 1 s long pure tone is played, followed by a variable 200–400 ms delay (300 ms on average, with uniform distribution), after which the animal receives water reward in 80% of the trials, in a pseudorandomized order.
  d. In this stage, there is no punishment; therefore, the reinforcer is omitted in the other 20% of the trials.

***Note:*** We recommend starting the training with the likely reward cue, since the high probability of reward after the cue motivates mice better during the initial training phase.

***Note:*** If the mouse licks in the foreperiod, the trial is restarted from the no-lick period. This way of enforcing no licking before the stimulus is not critical for forming the cue-reward association. However, it helps interpret the anticipatory lick response of mice (see quantification and statistical analysis). It also helps disentangle neural responses correlated with licking vs. different phases of the trials when concurrent recordings are performed (see problem 3).

**CRITICAL:** The variable delay between tone offset and reward delivery is important for the formation of the anticipatory lick response (see quantification and statistical analysis). In our experience, if the reward delivery time is fully predictable, some mice do not develop anticipatory lick responses.

4. Introducing the cue that will predict likely punishment (Stage 2)
  a. The animal performs 5 operant trials before the cued outcome trials. Two pure tones of well-separated pitch (e.g., 4 kHz and 12 kHz) predict likely reward (75% of all trials) or unlikely reward (25% of all trials) in a pseudorandomized order. (The latter cue will predict likely punishment in future training phases.)
  b. There is no punishment in this training phase; therefore, the likely reward cues are followed by reward in 80% and omission in 20% of the cases, whereas the unlikely reward cues are followed by reward in 25% and omission in 75%.

***Note:*** We recommend testing mice with reversed cue-reward contingencies to rule out that the cue tones themselves induce differential behavior or neural activity based on their potential behavioral significance or previous experience of the animals.

5. Introducing punishment (Stage 3)
  a. Operant trials are not included for this phase.
  b. Air puff punishments are introduced in this phase. This modifies the outcome probabilities as follows: the likely reward cues predict 80% reward, 10% punishment, 10% omission and the likely punishment cues predict 25% reward, 65% punishment and 10% omission.
  c. These probabilities represent the final task contingencies. The two trial types are pseudorandomly interleaved in a 3:1 ratio, as in the previous phase.

***Note:*** The animals’ performance can transiently drop at this stage due to the aversive nature of air puff punishment (see problem 4).

6. Ramping up likely punishment trials (Stage 4)
  a. Likely reward and likely punishment trials are pseudorandomly interleaved in a 6:4 ratio.
7. Final task (Stage 5)
  a. Likely reward and likely punishment trials are pseudorandomly interleaved in a 1:1 ratio.
  b. This is the final phase of the training protocol, and it can be maintained throughout the course of the experiment.

***Note:*** Mice are actively engaged in more trials if they are trained at the beginning of their active (dark) phase (Remmelink et al., 2017; Birtalan et al., 2020), although we had success in training during the light phase as well. Nevertheless, we recommend adjusting the time of training to the light/dark cycle of the animals (see also problem 5).

**CRITICAL:** Training should be performed at a consistent time of day, maintained throughout the experiment (Gritton et al., 2012).

***Note:*** When training multiple animals sequentially, keeping the order of mice helps maintain consistency during the experiment and further reducing environmental factors that can lead to poor performance. However, see problem 4.

**CRITICAL:** Overtraining animals on intermediate stages slows down or prevents further learning; ideally do not train more than 1 or 2 days on the intermediate stages of the task. Note that aged (over 12 months) mice learn slower and may be trained up to 3 days on intermediate steps.

**CRITICAL:** Skipping training days at the initial phase of the training (especially in the first week) can significantly reduce performance and the animal may not be able to learn the task eventually.

**CRITICAL:** The cumulative water consumed during the training session should be taken into account when calculating daily water consumption.

**CRITICAL:** The daily water should be provided hours after the training session, ideally approximately at the same time every day, to prevent mice from forming an association between water and the end of training.

### Perfusion

**Timing: 1 h**

After concluding the behavioral experiments, animals are anesthetized, and the brain tissue is preserved by transcardial perfusion of 4% paraformaldehyde (PFA) fixative.

***Note:*** This process is performed when the training protocol is combined with neuronal recordings or optogenetic manipulations, to allow histological processing of the brains. However, transcardial perfusion is not necessary for purely behavioral or systemic pharmacological manipulation studies.

8. Put mice in an isoflurane chamber to induce anesthesia.
9. Inject mice with a mixture of ketamine, xylazine and promethazine intraperitoneally (83 mg/kg, 17 mg/kg and 8 mg/kg respectively, dissolved in saline) to achieve deep anesthesia.
10. Wait until the animal does not respond to tail pinch.
11. Restrain the limbs on the perfusion platform.
12. Start the perfusion pump with saline.
13. Make a longitudinal cut on the skin over the abdominal region by using small surgical scissors and tweezers.
14. Hold the xiphoid process with a pair of forceps and cut the diaphragm to open the chest cavity. Carefully avoid piercing the heart.
15. Cut the ribcage on both sides; hold the basis of the sternum and lift it up, exposing the heart.
16. Make a small incision at the apex of the left ventricle and insert a feeding needle (connected to the perfusion pump). Gently push the needle into the aorta. Fix the needle with a small clamp.
17. Make a small cut on the right atrium to drain the blood.
18. Perfuse with saline for ∼2 min, then switch to 4% paraformaldehyde fixative for an additional 20 min.
19. Stop the pump and disconnect the feeding needle.
20. Decapitate the animal and remove the brain.

***Optional:*** The brain can be immersed in 4% PFA for enhanced preservation or can be stored in a buffer solution with sodium-azid at 4C°.

## Expected outcomes

During successful training, mice form an association between the outcome predicting cues and the reinforcers. They learn the task contingencies, which is expressed by their differential anticipatory licking in response to the likely reward and likely punishment predicting cues (see quantification and statistical analysis). We consider a licking response anticipatory when it happens after the cue onset but before reinforcement delivery (∼1.2 s time window from cue onset in the present protocol). Note that for learning outcome probabilities, mice have to integrate rewards over multiple trials. Therefore, this is a harder task than associating specific cues with different reward size, which can be inferred from a single trial (Stephenson-Jones et al., 2020).

## Quantification and statistical analysis

**Figure 4:**
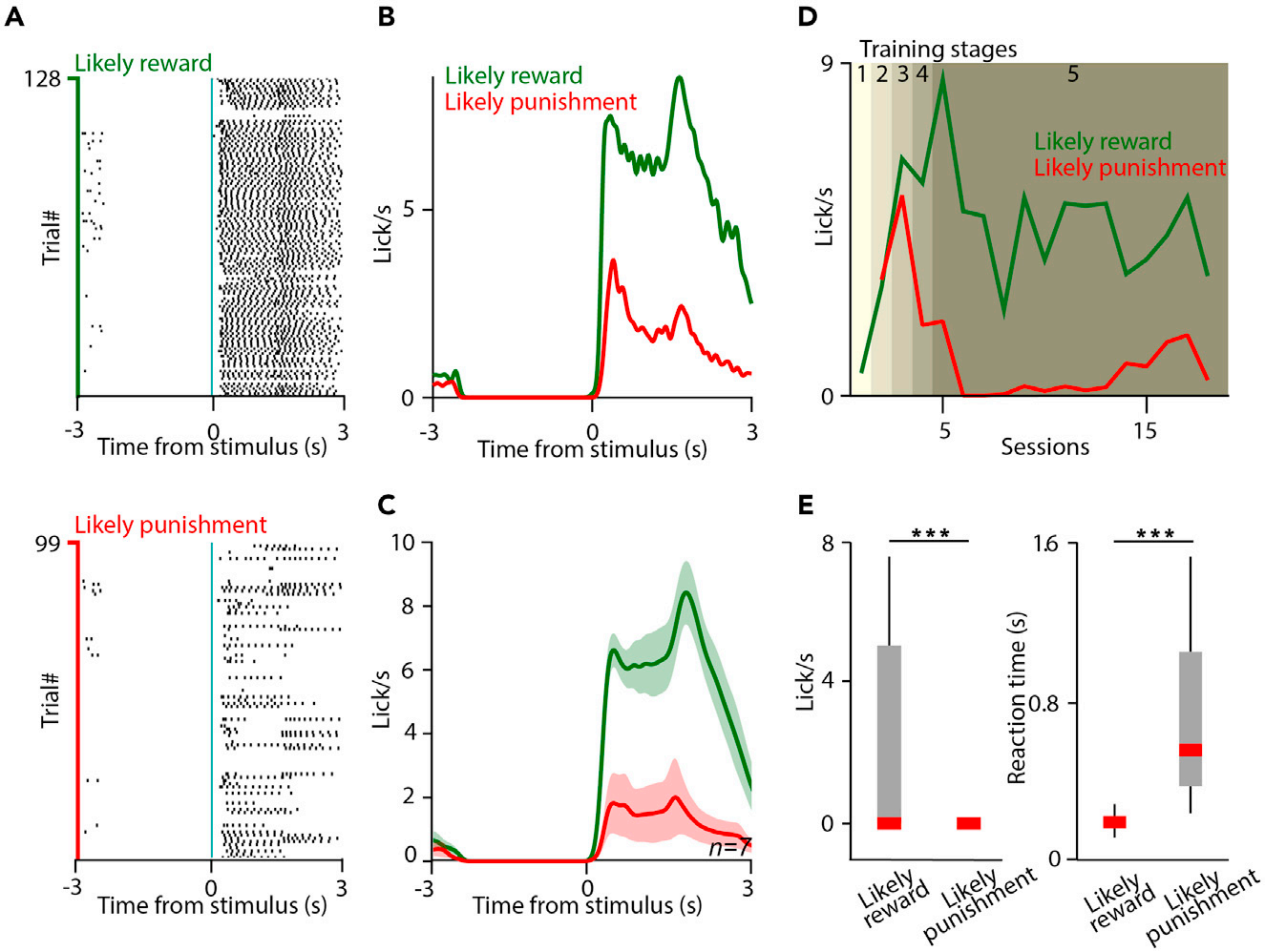
Behavioral analysis (A) Raster plot of individual licks aligned to cue onset from an example session. Top, licks after the cues predicting likely reward; bottom, licks after the cues predicting likely punishment. (B) Peri-event time histogram (PETH) of licking frequency aligned to cue onset corresponding to the same example session. (C) Average PETH of anticipatory licking of n = 7 training sessions. Error shade represents SEM. (D) Learning curve of an example mouse. (E) Statistical comparison of lick rate (left) and reaction time (right) after cues predicting likely reward or likely punishment in an example session with n = 165 likely reward trials and n = 165 likely punishment trials. For reaction time comparison, the trials in which mice showed licking were used (n = 80 likely reward trials and n = 8 likely punishment trials). Box-whisker plots show median, interquartile range and non-outlier range. ∗∗∗, p < 0.001, Mann-Whitney U test.

We suggest that simple behavioral analysis should be performed on each training day to monitor the progress of the animals during training. Mice start licking the water spout after cue presentation in the anticipation of reward. As training progresses and mice start learning the cue contingencies, they develop differential anticipatory licking in response to the cues: they lick more vigorously (with higher frequency) if reward is more likely. We routinely calculate and visualize lick rasters (Figure 4A) and peri-event time histogram of licking activity (Figures 4B and 4C) aligned to cue onset to judge the difference in anticipatory lick rate after the sound cue. Learning curves can be plotted by tracking the changes in anticipatory lick rate across training days (Figure 4D). Reaction times can be measured as the time difference between cue onset and the first lick of the animal (Figure 4E). We use the custom-developed ultimate_psth.m Matlab code for calculating anticipatory lick rates, available at https://github.com/hangyabalazs/CellBase.

## Limitations

This training protocol allows efficient training of mice on a probabilistic Pavlovian task, enabling direct control over outcome contingencies. Nevertheless, it has some limitations.

Training multiple animals in parallel can be time-consuming and labor-intensive, limiting the number of mice. This could be solved by fully automating the training (Qiao et al., 2019; Birtalan et al., 2020); however, that would either require using voluntary head-fixation protocols (Scott et al., 2013; Aoki et al., 2017) or switching to a freely behaving paradigm.

Efficient training requires some level of specific expertise from the experimenter. In our experience, the crucial skill is to learn the proper positioning of the lick port. While the feedback on behavioral performance from the Bpod behavior control system is useful, it is possible that the mouse can perform the task but stops early due to suboptimal positioning of the lick port. If this happens early in training or multiple times, it may influence performance on the long term. This could be improved by including a motorized stage for moving the lick port, which would allow reliable positioning and even enable automating this important step.

In Pavlovian conditioning tasks, the outcome does not depend on the animals’ actions, endowing the experimenter with full control over the outcomes. However, as a downside, it is hard to measure the animals’ performance and get access to the level of learning. We address this by using the difference in anticipatory lick rate that develops throughout the task (see quantification and statistical analysis). Nevertheless, there is no guarantee that a linear relationship exists between learning outcome probabilities and anticipatory licking, which complicates the interpretation of performance comparisons across mice.

## Troubleshooting

### Problem 1

The animal might show signs of strong distress (vocalizing and attempting to escape) at the first session that prevents starting the Operant phase (step 2).

### Potential solution

Remove the animal from the setup immediately to prevent further stress. Take a couple more days before the training, and while handling the animal, place it on the setup platform and let it explore. Consider giving it the daily amount of water on the setup platform as well. This way the experimenter can reduce the anxiety of the animal associated with head restraint.

### Problem 2

The animal does not lick for water in the Operant phase (step 2).

### Potential solution

Repeat the Operant phase at another time. Do not leave the mouse in the setup for an extended period, which often causes stress.

### Problem 3

The animal shows impulsive licking behavior that interferes with the training. If a no-lick period is used in the protocol, this behavior leads to multiple restarting of most trials (step 3).

### Potential solution

A brief period of free water before or at the beginning of the training session may help reduce impulsive licking. Counterintuitively, larger reward sizes can lead to impulsive/poor behavior (Kuchibhotla et al., 2019). We suggest starting from a reward size of 5 μL at the beginning of training and decreasing it gradually to 2–3 μL for best performance.

### Problem 4

The mouse stops licking upon the introduction of punishment (step 5).

### Potential solution

The first introduction of the punishment during training (Stage 3) might cause a setback in the animal’s performance. First, repeat Stage 3 on the next day. If the problem persists, it might help training one or two more days at Stage 2; however, overtraining on intermediate phases should generally be avoided. As a rule of thumb, advance the mouse to the next stage as soon as it responds to at least 30% of the likely reward cues.

### Problem 5

The animal stops licking early during the training session (step 7).

### Potential solution

Allowing some free water by briefly opening the water valve via Bpod might help increase motivation. When training multiple animals sequentially, training at a slightly different time of day by changing the order of mice may help optimize their individual performance. However, try this only if problems occur; otherwise keeping the same order of mice helps develop consistent performance.

## Resource availability

### Lead contact

Further information and requests for resources and reagents should be directed to and will be fulfilled by the lead contact, Balázs Hangya (hangya.balazs@koki.hu).

### Materials availability

This study did not generate new unique reagents.

## Data Availability

MATLAB code developed to analyze the data is available at www.github.com/hangyabalazs/VP_data_analysis. Behavioral data is available from the technical contact upon reasonable request.
